# Retesting The Validity Of A Specific Field Test For Judo Training

**DOI:** 10.2478/v10078-011-0048-3

**Published:** 2011-10-04

**Authors:** Luis Santos, Vicente González, Marta Iscar, Juan I. Brime, Javier Fernández-Río, Blanca Rodríguez, Mª Ángeles Montoliu

**Affiliations:** 1 Medical Service of the Community of Cabo Peñas, Luanco, Spain; 2 Medical Service of the Community of Cabo Peñas, Luanco, Spain; 3 Exercise Physiology Unit, Hospital Universitario Central de Asturias, Spain; 4 Department of Functional Biology, University of Oviedo, Spain; 5 Department of Educational Sciences, University of Oviedo, Spain; 6 Exercise Physiology Unit, Hospital Universitario Central de Asturias, Spain

**Keywords:** Aerobic-anaerobic transition, combat-sports, physiological demands

## Abstract

The main goal of this research project was to retest the validity of a specifically designed judo field test (Santos Test) in a different group of judokas. Eight (n=8) national-level male judokas underwent laboratory and field testing. The mean data (mean +/− SD) obtained in the laboratory tests was: HR_max_: 200 ± 4.0 beats × min^−1^, VO_2 max_: 52.8 ± 7.9 ± ml × kg^−1^ × min^−1^, lactate max: 12 ± 2.5 mmol × l^−1^, HR at the anaerobic threshold: 174.2 ± 9.4 beats × min^−1^, percentage of maximum heart rate at which the anaerobic threshold appears: 87 ± 3.6 %, lactate threshold: 4.0 ± 0.2 mmol × l^−1^, and RPE: 17.2 ± 1.0. The mean data obtained in the field test (Santos) was: HR_max_: 201.3 ± 4.1 beats × min^−1^, VO_2 max_: 55.6 ± 5.8 ml × kg^−1^ × min^−1^, lactate max: 15.6 ± 2.8 mmol × l^−1^, HR at the anaerobic threshold: 173.2 ± 4.3 beats × min^−1^, percentage of maximum heart rate at which the anaerobic threshold appears: 86 ± 2.5 %, lactate threshold: 4.0 ± 0.2 mmol × l^−1^, and RPE: 16.7 ± 1.0. There were no significant differences between the data obtained on both tests in any of the parameters, except for maximum lactate concentration. Therefore, the Santos test can be considered a valid tool specific for judo training.

## Introduction

Jigoro Kano, the father of judo, defined it as having three stages. At “low-level judo”, judokas practice offensive and defensive techniques in *randori* and *kata*. At “mid-level judo”, judokas grow physically, but also mentally. At “upper-level judo”, judokas benefit the society with their spirit of “maximum efficiency” using their strength of body and mind (Shishida, 2010). Today, judo is one of the most important combat Olympic sport disciplines.

The physiological demands of judo have been difficult to establish. Judokas compete in different categories according to their weight, so the anthropometric variability among competitors is very high. The technical-tactical level of the athletes or the specific technical actions performed during contests can also be very diverse. The wide range of combats’ length (from seconds to minutes), and the existence of a series of rest-activity cycles, increases that variability. Moreover, judokas can take part in several combats during the same day within a short period of time. Therefore, it is difficult to quantify the effort displayed by an athlete during a contest (Majean and Galliat, 1986). Verkhoshansky (2002) believes that judo is a sport that requires a specific resistance capacity, since the competition takes place at an intermittent work rate. Furthermore, Pulkkinnen (2001) considers that the anaerobic metabolism is the primary source of energy in judo combat. However, Thomas et al. (1989) think that judokas also need adequate aerobic endurance to keep their performance at a high level throughout the whole combat. Based on these ideas, Wasserman and Mcllroy (1964) launched the notion of the anaerobic threshold: a progressive shift from oxidative to anaerobic metabolism. The aerobic-anaerobic transition zone is the key factor to develop the aerobic capacity and the aerobic power of athletes and, as stated earlier, judo combat demands a high level of energy production from both systems. Moreover, Laskowski et al. (2008) verified that judo training improves both aerobic and anaerobic performance.

Previous research (Santos et al., 2010) showed the Santos test as an easy, explicit, valid, consistent and reproducible method for determining the aerobic-anaerobic transition zone in competitive judokas. It mimics real judo competition, which makes it a great tool for judo training. It has been considered a useful tool to design and assess training protocols for high-level judokas.

In the present study, we attempted to retest the validity of the Santos test using a different group of high-level judokas. Our working hypothesis stated that if the results from the laboratory test and the field test (Santos) are similar, it can be considered a valid tool for any judoka.

## Methods

### Participants

Eight (n=8) high-level male judokas volunteered to participate in this study. Prior to the beginning of the investigation, all procedures were approved by the Bioethics Committee of the Central University Hospital of Asturias (Spain) according to the declaration of Helsinki. Subjects were informed of the experimental risks and they all signed a written consent. In order to participate in this study, subjects had to fulfil certain requirements: they had to be black belt first Dan (Master degree in judo), and they had to be regional champions and medallists in national championships (high-level athletes). The anthropometric variability among competitors is very high in judo (Franchini et al., 2011). Therefore, we selected one subject for each (under-23) male weight categories (−60 kg, −66 kg, −73kg, −81 kg, −90 kg, −100 kg, +100 kg) to avoid the influence of the subjects’ weight on the results of the investigation. Table 1 presents the main characteristics of the judokas who took part in the study.

### Procedures

Our assessment procedure included two parts: a laboratory test and a field test. Both followed a progressive interval maximal protocol. Moreover, the field test (Santos test) was designed to match the laboratory test’s main features.

As stated earlier, previous research has shown that it complies with the principles of validity, specificity, individuality and reproducibility (Santos et al., 2010). The main goal was to be able to compare results from both assessment tools. All subjects were required to stay away from any type of exercise 24 hours before each testing session. The time lapse between field and laboratory tests was always less than 7 days. Participants were familiarized with all the procedures, and before each test, they performed the same standard warm up.

### Laboratory test

A standard treadmill (Laufergotest LEB, Germany) was used to carry out the test. Special environmental measures were implemented to ensure proper ventilation. Meteorological conditions were kept constant throughout the whole trial period (temperature: 17–20°C, atmospheric pressure: 730–740 mm Hg). A standard protocol, reflecting the generally accepted recommendations for evaluating VO_2_ and/or HR in 3-minute work steps, was followed (ACSM, 2000): initial velocity: 5 km × h^−1^, velocity increments: 2 km × h^−1^, effort stages: 3-minutes, treadmill inclination: 5% (constant), and pause: 30-second between stages. This type of SPIM test is widely used in sport research (Gullstrand et al., 1994). Stegman and Kinderman (1982) recommend a running protocol of 3 minutes per stage with an intensity increment of 2 km × h^−1^ until exhaustion as the best approach to determine the individual anaerobic threshold (IAT). Furthermore, heart rate and maximum oxygen uptake stabilize within this 3-minute time frame (Chicharro et al., 1997). Respiratory datawas recorded using a CardioO_2_ & CPX/D gas analyzer (Medgraphics, USA). The oxygen analyzer was zirconium, while the carbon dioxide analyser was infrared. The ventilation was measured with a Hans-Rudolph mask fitted with a Pittot pneumotachograph calibrated before and after each test. The average values were measured breath by breath, and calculated each 30 seconds. The peak value was the highest of all the averages obtained. Continuous electrocardiogram analyses were carried out to measure the subjects’ heart rate. Blood pressure was also measured with a precision mercury sphygmomanometer. Blood lactate was analyzed using an Accusport (Boehringer, Germany) apparatus (Dascombe et al., 2007). Gullstrand et al. (1994) investigated whether lactate concentrations in blood obtained from an incremental test on a treadmill vary when the blood sample is extracted during a 30-second rest period compared to a continuous effort. Their results showed that no step of the test yielded statistically significant differences between the average lactate values in capillary blood or in heart rate. In addition, Beneke et al. (2002) investigated whether the interruptions needed to draw blood samples during a constant effort test had any impact on the blood lactate concentrations in blood, on maximum lactate steady state, the effort at the maximum lactate steady state, or on the relative work intensity at the maximum lactate steady state. They found that such interruptions of the workload (30 and 90 seconds) lead to a decrease in the lactate value in blood only after a 30-minute work period. None of our laboratory tests lasted more than 30 minutes. Therefore, the pauses required to collect blood samples did not affect lactate concentration. The following parameters were evaluated: (1) maximum heart rate (HR_max_); (2) heart rate at the anaerobic threshold (HR threshold); (3) percentage of threshold heart rate with respect to the maximum (HR threshold %); (4) maximum oxygen uptake (VO_2max_) measured in ml × kg^−1^ × min^−l^; (5) concentrations of basal blood lactate at the end of each effort stage and 3 minutes after the conclusion of the test (mmol × l^−1^); (6) lactate maximum (mmol × l^−1^); (7) maximum speed attained by the subjects on the treadmill (km/h). The criteria employed to determine the realization of the maximum effort was: (1) respiratory quotient (RQ) ≥ 1,15; (2) HR ≥ 85% of the theoretical value. Lastly, Franklin et al. (2000) criteria were applied to finish the test.

### Field test

It was designed to create an assessment tool that could mimic real competition conditions. Thus, it was implemented on a tatami (competition judo floor). Two judokas were required to carry out the test: the subject and a supporting partner. Both of them belonged to the same weight category, and they were dressed in judo suits (judogi). During the test, each subject had to perform several sequences of three specific technical skills; the ones that he performs better and uses during competition (tokui-waza or special technique). Each sequence had 2 parts: (1) active phase: the judoka had to perform the specific technical skills without bringing the opponent to the floor. It lasted 40 seconds. The dependent variable was the number of repetitions carried out by the subject, which produced a progressive increase in the intensity of the trial. Three different technical skills were performed in successive sequences. In the first one, the subject raised his partner from the floor. In the second one, he unbalanced completely his opponent. Finally, in the third one, the subject chose to lift the opponent or unbalance him completely. Thus, we created a format that was repeated throughout the whole test: technique one performed in the first 40-second phase, technique two in the second 40-second phase and technique three in the third 40-seconds phase. The next cycle started again with the first technique, and continued with the same sequence until exhaustion. (2) Passive phase: the judoka and his supporting partner, grabbing each other with their hands (both judokas have to show the right or left basic judo hold - kumikata) had to move from one side to the other of the tatami depicting a square on it: first, towards the left of the supporting partner; second, backwards; third, towards the right of the supporting partner, and fourth, forward to the starting point. This phase tried to match the displacements that take place in real combats. It lasted 15 seconds, and it was performed right after every active phase (40 seconds). It gave the test its intermittent character, a key element in judo training and combat.

The progressiveness of the test was based on the increase of one repetition on each new 40-second series. The first active phase started with 7 repetitions, the 2^nd^ had 8, the 3^rd^ had 9, and so on, until exhaustion prevented the judoka from executing the specific technical skill with the required quality, which followed these rules: (1) the judoka was not able to raise his partner from the floor, (2) he could not throw his partner off balance, and/or (3) he could not complete the correct number of repetitions in 40 seconds. Most studies refer to judo combat as several intervals of activity and pause with a regular character (Favre-Juvin et al., 1989; Thomas et al., 1989; Pulkkinen, 2001; Sbriccoli et at., 2007; Franchini et al., 2009b). That is the reason why we divided the field test in two phases (activity and pause) that take place at a regular pace. Favre-Juvin et al. (1989) reported activity periods of 20 to 40 seconds with interruptions from 10 to 20 seconds. Therefore, we estimated that the best rate activity-pause was 40-15 seconds, respectively. Our goal was to match, as closely as possible, the real effort displayed by judokas in competition.

During the field test, the judokas wore a VO_2_ 2000 portable gas analyzer (Med Graphics, USA) that has been proven a valid tool for field assessment of oxygen uptake (Yeo et al., 2005). The dimensions of this system were: (1) length: 11 cm; (2) width: 14 cm; (3) thickness: 6.5 cm; (4) weight: 1200 grams. It was calibrated before and after each test with Aerograph software (Windows 95 and 98 compatible). The ergospirometric parameters studied were oxygen uptake, carbon dioxide production, ventilation and respiratory coefficient. Heart rate was continuously recorded by means of a heart rhythm monitor (Polar S810, OY Finland), and it was digitalized using a Digital Wireless Industrial Transceiver (model Wit 2410 E, 2.4 GHz). Throughout the test, micro samples of arterialized blood were obtained from perforation of the earlobe in order to determine the blood concentrations of lactate. The samples were taken before the test, when the ventilation threshold was being reached (as determined from the data obtained in real time from the portable gas analyzer), and 5 minutes after the end of the test. The test was considered terminated when the athletes could no longer meet the previously indicated quality requirements. The blood lactate was analyzed with the same equipment and procedure used in the laboratory test.

In order to verify the validity of our proposal, the same parameters were studied in the field and laboratory tests. Each subjects’ IAT was measured using Keul et al.’s procedures (1979). These researchers considers that the workload, the VO_2_ or the treadmill velocity corresponding to the point cut by a tangent on the lactate curve with an angle of 51º represents the IAT of the subject.

### Rating of Perceived Exertion (RPE)

Morgan and Borg (1976) observed that the rate of change in the rating of perceived exertion (RPE) during prolonged work can be used as a sensitive predictor of the point of self-imposed exhaustion. The commonly employed Borg 6–20 scale assumes a linear function between perceptual and physiological (VO_2_, HR) or physical (work rate) parameters (Borg, 1998). Recently, the use of RPE has been applied to resistance training in an effort to create a valid, non-invasive way to monitor training intensity (Sweet et al., 2004). However, the existing literature on RPE applied to judo athletes is scarce.

The subjects were given standardized instructions on how to implement the scale during their test session. The scale remained in full view of the judokas for the duration of the test. They were asked to rate their perceived exertion on the Borg’s scale at the end of the trial. Mean and standard deviation were calculated, and values were classified in accordance with the American College of Sports Medicine qualitative descriptors.

### Statistical analysis

All statistical analyses were carried out using the SPSS 12.0 programme for Windows, applying the Student’s *t*-test, and considering the minimum level of significance as p<0.05. This procedure was used because it has been considered valid to compare different performances of the same subject (Montoliu et al., 1997). In order to verify whether the differences between the mean data on laboratory and field tests were statistically significant, and to show the reproducibility of the field test, we performed the statistical T-test, also known as Student’s *t*-test.

## Results

The mean data obtained in the laboratory tests was: HR_max_: 200 ± 4.0 beats × min^−1^, VO_2 max_: 52.8 ± 7.9 ± ml × kg^−1^ × min^−1^, lactate max: 12 ± 2.5 mmol × l^−1^, and RPE: 17.2 ±1.0 (Table 2). The mean data obtained in the field (Santos) test was: HR_max_: 201.3 ± 4.1 beats × min^−1^, VO_2 max_: 55.6 ± 5.8 ml × kg^−1^ × min^−1^, lactate max: 15.6 ± 2.8 mmol × l^−1^, and RPE: 16.7 ±1.0 (Table 2). Table 2 also shows the application of the statistical t-test to mean values obtained in the two tests. The differences between the main data of both tests were not statistically significant in most of the parameters studied. The only exception was the maximum lactate concentration.

The mean data corresponding to the aerobic-anaerobic transition zone of the subjects obtained through the laboratory tests was: HR at the anaerobic threshold: 174.2 ± 9.4 beats × min^−1^, percentage of maximum heart rate at which the anaerobic threshold appears: 87 ± 3.6 %, lactate threshold: 4.0 ± 0.2 mmol × l^−1^. Similarly, the data obtained through the field (Santos) test was: HR at the anaerobic threshold: 173.2 ± 4.3 beats × min^−1^, percentage of maximum heart rate at which the anaerobic threshold appears: 86 ± 2.5 %, lactate threshold: 4.0 ± 0.2 mmol × l^−1^. The differences between the data of both tests were not statistically significant.

## Discussion

The present study has proven, again, the Santos test as a simple, specific, valid and reliable field test for judo. In addition, it has shown several important physiological parameters of high-level male judokas (maximum heart rate, maximum oxygen consumption, maximum lactate, heart rate at the anaerobic threshold, lactate at the anaerobic threshold), as well as the rating of perceived exertion, obtained through laboratory and field tests.

Regarding maximum heart rate, our group of male judokas showed a mean value of 200 ± 4.0 beats × min^−1^ in the laboratory test. Thomas et al. (1989) recorded a maximum value of 191 beats × min^−1^ in a group of judokas of the Canadian senior national team. The results of both studies are very similar.

In the field (Santos) test, our subjects showed mean values of 201.3 ± 4.1 beats × min^−1^. Baudry and Roux (2009) reported values of 193.9 +/− 7.0 beats × min^−1^ in a group of 10 subjects (2 girls and 8 boys) performing a judo-specific circuit training session. Houvenaeghel et al. (2005) obtained a HR_max_ value of 196 beats × min^−1^ during an intermittent effort based judo exercise. Franchini et al. (2007) reported values of 181 ± 10 beats × min^−1^ in elite judokas, and 186 ± 11 beats.min^−1^ in non-elite judokas using a special judo fitness test.

Our field test (Santos) was designed to imitate judo-competition. Therefore, its results should be similar to the ones obtained in real competition. Sanchis et al. (1991) recorded mean maximum heart rates of 198 beats × min^−1^ in a competition among regional-level judokas, while Deugotte et al. (2003) obtained a HR_max_ value of 182.4 ± 0.2 beats × min^−1^ during several judo combats with national-level judokas. As stated in the introduction, we can see how difficult it is to obtain standard physiological parameters in judokas due to the specific features of judo competition. Moreover, the Golden Score Rule adds new difficulties to this task. If the scores of both competitors are identical at the end of a bout, the contest is solved through the Golden Score rule. This is a sudden death situation where the clock is reset to fight-time, and the first contestant to achieve any score wins. If there is no score during this period, the winner is decided by Hantei (decision) of the referee and the two corner judges. Boguszewski (2011) has showed that the number of fights that used the golden point is increasing from year to year in the last top world judo male tournaments. Nevertheless, according to Spanish Judo Federation data, the Golden Score Rule appeared in only 11 out of 175 combats in the 2011 Under-23 Spanish National Judo Championship. According to Boguszewski (2011), the Golden Score Rule extends the combat time, and the longer the judo combat, the more aerobically dependent it becomes. Therefore, it indicates the need to study the aerobic-anaerobic transition zone in judo, and the Santos Test responds to this necessity.

In our study, there were no significant differences between the mean HR_max_ data obtained in the laboratory and field tests. These results allow us to assert that, in both cases, the judokas’ effort was maximal. Thus, the heart rate obtained in the athletes at the end of both tests could be considered their maximum.

Regarding VO_2max_, our subjects achieved values of 52.8 ± 7.9 ml × kg^−1^ × min^−1^ in the laboratory test. The existing literature (Favre-Juvin et al., 1989; Thomas et al., 1989; Callister et al., 1990; 1991; Ebine et al., 1991; Sterkowicz, 1995; Franchini et al., 2007; Sbriccoli et al., 2007) show values in similar laboratory tests (treadmill performance) ranging from 47.9 to 62.6 ml × kg^−1^ × min^−l^ in high-level judokas. These results confirm that our subjects have VO_2max_ levels similar to those of the elite. Other results, like the ones obtained by Baudry and Roux (2009) cannot be compared with ours because these researchers used a different testing procedure (their subjects were asked to cycle on an electronically braked ergometer and the results were; 53.3 ± 8.6 ml × kg^−1^ × min^−1^).

In the field (Santos) test, our subjects achieved a VO_2max_ value of 55.6 ± 5.8 ml × kg^−1^ × min^−1^. In a previous study (Santos et al., 2010), a different group of high-level male judokas reached VO_2max_ values of 59.8 ± 3.6 ml.kg^−1^ × min^−1^. Two analogous studies carried out in two different groups of subjects have yielded similar results. To our knowledge, there are no other published works that have studied maximum oxygen uptake in judokas in a field test.

VO_2max_ results from the laboratory and field tests were not significantly different. Consequently, the subjects’ effort on both tests was similar, and they could be considered maximal.

Concerning the concentration of blood lactate, our judokas achieved values of 12 ± 2.5 mmol × l^−1^ in the laboratory test. Thomas et al. (1989) recorded a mean 15.2 mmol × l^−1^ of lactate in Canadian judokas in a similar test.

When we conducted the tests on the tatami (field test), the value obtained was 15.6 ± 2.8 mmol × l^−1^. Previous studies have reported values ranging from 6.4 to 17.9 mmol × l^−1^ (Sikorski et al., 1987; Sanchis et al., 1991; Drigo et al., 1995; Heinisch, 1997; Serrano et al., 2001; Franchini et al., 2003; Sbriccoli et al., 2007; Braudry and Roux, 2009; Franchini et al., 2009b). Unfortunately, different testing procedures with different protocols (judo-specific circuit training exercises, special judo fitness test) have yielded a wide variety of results. Nevertheless, when the field test was a real competition or a practice combat the results increased to a higher range: 9 to 20 mmol × l^−1^ (Sanchis et al., 1991; Drigo et al., 1995; Serrano et al., 2001; Sbriccoli et al., 2007). The field test used in this study (Santos) was designed to mimic real competition conditions, and all of our subjects achieved values within this range. This fact reaffirms the idea that the Santos test is an adequate tool to improve judokas’ performance in competition.

Besides, maximum blood lactate reached 15.6 ± 2.8 mmol × l^−1^ in our field test. This value is significantly higher than the one obtained in the laboratory test. This is possible because of the greater muscular involvement required in the field test. Judo combat recruits more muscle fibers (whole body) than running on a treadmill (legs). Therefore, a higher lactate acid production should be expected.

Regarding the IAT, male judokas undergoing laboratory tests (Gorostiaga, 1988) manifest it at 4 mmol × l^−1^ of lactate concentration, and at a running speed of 9–13 km × h^−1^ (depending on the physical condition of the athlete). Our male judokas reached their IAT at 174.2 ± 9.4 beats × min^−1^, which is equivalent to 87 ± 3.6 % of HR_max_, a lactate concentration of 4.0 ± 0.2 mmol × l^−1^, and a running speed of 11–15 km × h^−1^. In another group of judokas (7 males and 1 female), Bonitch et al. (2005) found IAT values of 174 ± 9 beats × min^−1^, which are very similar to our results. In our field test, all judokas manifested their IAT between 12 and 15 repetitions, at a heart rate of 173.2 ± 4.3 beats × min^−1^, which is equivalent to 86 ± 2.5 % of HR_max_, and a lactate concentration of 4.0 ± 0.2 mmol × l^−1^. Therefore, no significant differences were observed between the values obtained in the laboratory and in the field test. In a previous study (Santos et al., 2010), a different group of high-level male judokas reached their IAT in the laboratory test at 170.3 beats × min^−1^ (85.9% of HR_max_), and in the field test between 11 and 15 repetitions and at a heart rate of 169.7 beats × min^−1^ (85.0% of HR_max_). Therefore, two analogous studies carried out in two different groups of subjects have yielded similar results. To our knowledge, there are no other published works that have studied judokas’ IAT on field tests.

The subjects’ mean RPE was 17.2 ± 1.0 in the laboratory test, and 16.7 ± 1.0 in the field test. Once again, there were no significant differences between both tests. Both results are very high, and reinforce the idea that all subjects performed both tests at maximum intensity. To the researchers’ knowledge, only three published studies have measured RPE in judo. Bonitch et al. (2005) obtained a value of 19 ± 1 in a group of young judokas (7 males and 1 female) at the end of an incremental laboratory test (treadmill). Arruza et al. (1996) reported mean maximum values of 13.7 after a judo training combat (simulating real competition conditions) in a group of 65 Olympic judokas (32 women and 33 men). Similarly, Serrano et al. (2001) obtained mean maximum values of 14.6 (*SEM*=0.6; range: 11 to 18) in a group of 13 judokas after a series of 3 judo training combats (simulating real competition conditions). These results are very similar to the one obtained in our field test (16.7 ± 1.0). It reinforces the idea that the Santos test’s features are comparable to judo competition. Therefore, it is a good tool to improve judo performance in competition.

Concerning the subjects’ cardiac response to a progressive effort, our field test also fulfilled Conconi’s requirement: a break-off in the ascendant regularity of effort means the beginning of the aerobic-anaerobic transition zone of the subject under assessment (Conconi et al., 1996). Consequently, the Santos test could be used by coaches as a simple and non-invasive tool to determine the specific and individual aerobic-anaerobic transition zone of their judokas. They just have to apply the test and use a heart-rate meter to control the cardiac response of the judokas. This parameter has already been considered a specific reference for judo training by Sterkowicz (1995) and other authors (Franchini et al., 2009a; Sterkowicz-Przybycień, 2009; Boguszewska et al., 2010). Hence, it has been disclosed as useful for prescribing specific judo training tasks.

The Santos test can be considered specific and individual because: (1) it is based on the use of the specific technical skills of the judoka; (2) effort and rest times match those indicated by previous researchers for real combats (Gorostiaga, 1988; Favre-Juvin et al., 1989); (3) it follows the reliability principle (Santos et al., 2010); (4) laboratory data and Santos test data are very similar.

Moreover, Boguszewski (2011) believes that the Golden Score rule determines judokas’ effort during combat. This regulation makes combats last longer and places more demands on the aerobic system of the athlete. Therefore, it is even more important to determine the aerobic-anaerobic transition zone of the competitor to develop specific training tasks. The Santos test has been specifically designed to provide information on this specific parameter.

In conclusion, previous research has shown the Santos test as a valid, specific, field test to assess and manage conditioning in male judokas. This study has demonstrated that it has the same qualities when it is applied to a different group of subjects. Thus, coaches could use the Santos test to help improve judokas’ performance in competition.

## Figures and Tables

**Table 1:** Anthropometric characteristics of the subjects (n=8)

Age (year)	19.7 ± 1.9
Body Mass (kg)	73.8 ± 13.3
Body Height (cm)	175.6 ± 3.9
Body fat (%)	11.2 ± 2.2

Values are mean ± SD

**Table 2:** *Values obtained in the Lab test and the field test for retesting the validity of Santos Test*.

	HR_max_ (beats × min^−1^)	HR threshold (beats × min^−1^)	HR threshold (%)	Lactate max. (mmol × l^−1^)	Lactate threshold (mmol × l^−1^)	VO_2max_ (ml × Kg^−1^ × min^−1^)	RPE (6–20 scale)
Lab. Test (Mean & SD)	200 ± 4.0	174.2 ± 9.4	87 ± 3.6	12 ± 2.5	4.0 ± 0.2	52.8 ± 7.9	17.2 ± 1.0
Santos Test (Mean & SD)	201.3 ± 4.1	173.2 ± 4.3	86 ± 2.5	15.6 ± 2.8	4.0 ± 0.2	55.6 ± 5.8	16.7 ± 1.0
Significance	NS (p<0.514)	NS (p<0.791)	NS (p<0.520)	S (p<0.020)	NS (p<0.838)	NS (p<0.437)	NS (p<0.350)

HR_max_= maximum heart rate; HR threshold= heart rate in the anaerobic threshold; HR threshold %= anaerobic threshold with respect to the maximum heart rate; Lactate max= maximum lactic acid concentration; Lactate threshold= lactate in the anaerobic threshold; VO_2max_= maximum consumption of oxygen in relative terms; RPE= rating of perceived exertion
